# Secure Edge Offloading Strategy for IoT-Enabled Electromagnetic Sensing Systems

**DOI:** 10.3390/s26175520

**Published:** 2026-08-31

**Authors:** Tong Xue, Yang Xia, Xiaolong Chen

**Affiliations:** 1 College of Electronic and Information Engineering, Shandong University of Science and Technology, Qingdao 266590, China; tongxue@sdust.edu.cn; 2 Engineering Technology Research Center of Industrial Automation Anhui Province, Hefei University of Technology, Hefei 230009, China; yangxia@hfut.edu.cn

**Keywords:** electromagnetic sensors, IoT-based sensing, communication and computation

## Abstract

Internet of Things (IoT) devices equipped with electromagnetic sensors, such as millimeter-wave radars, generate massive amounts of computation-intensive and latency-sensitive sensing data. However, their limited computational capability and energy supply hinder real-time local processing. Edge computing can alleviate this limitation by offloading electromagnetic sensing tasks to nearby edge servers. Nevertheless, the broadcast nature of wireless channels exposes sensing data to eavesdropping, while the heterogeneity of multi-source electromagnetic sensing data further complicates sensing-data offloading optimization. To address this issue, this paper develops secure edge-assisted sensing-data offloading framework for heterogeneous electromagnetic sensors deployed on IoT devices. Considering the energy consumed by electromagnetic sensing, wireless transmission, and edge computing, together with the end-to-end latency constraint, a secrecy energy efficiency maximization problem is formulated by jointly optimizing spectrum assignment, transmit power, and computing resource allocation. The simulation results demonstrate that the proposed algorithm improves secrecy energy efficiency at least 31% compared with benchmark schemes within a specific range, demonstrating its effectiveness for secure and energy-efficient electromagnetic sensing-data offloading.

## 1. Introduction

With recent advances in microwave and millimeter-wave technologies, electromagnetic sensors have been increasingly integrated into Internet of Things (IoT) devices for applications such as human activity recognition, contactless vital-sign monitoring, industrial inspection, structural health monitoring, autonomous driving, and environmental perception [1,2]. In particular, millimeter-wave radars can provide range, velocity, angle, and micro-Doppler information while maintaining robustness under adverse lighting and environmental conditions. These features make them promising sensing components for intelligent IoT systems [3,4].

The increasing sensing resolution, sampling rate, antenna-channel number, and signal-processing complexity of modern electromagnetic sensors, however, result in large volumes of computation-intensive and latency-sensitive sensing data. Extracting useful information from raw radar echoes may involve range and Doppler processing, beamforming, point-cloud generation, target detection, feature extraction, and intelligent classification. Such operations impose substantial computation and energy demands on resource-constrained IoT devices [5,6,7]. Local processing alone may therefore be unable to satisfy the stringent latency, accuracy, and energy-efficiency requirements of real-time electromagnetic sensing applications [8,9,10]. Cloud computing can provide abundant computational resources, but transmitting sensing data to remote cloud centers may introduce excessive communication latency and backhaul traffic. Edge computing provides a promising approach to address these limitations by deploying computing resources close to sensing devices [11].

### 1.1. Related Work and Motivation

Through computation offloading, an IoT device can transmit part or all of its electromagnetic sensing tasks to a nearby edge server for processing. This approach can reduce local computation pressure, shorten response time, and extend device operating life. For the partial sensing-data offloading, Liang et al. [12] designed an edge-based data sensing and offloading framework, and then optimized offloading decisions, bandwidth allocation and transmit power to reduce task completion latency and energy consumption. Furthermore, Yang et al. [13] proposed a cloud-enhanced-edge–end framework, and designed a sensing-data offloading algorithm based on a deep Q-network to dynamic channel conditions and time-varying computational workloads. For sensing-data offloading, in [14], the authors designed a edge computing network, where idle devices were applied to relieve computing burden on edge servers. Then, ref. [14] proposed a joint communication (i.e., power and spectrum) and computation (i.e., CPU frequency) resources strategy to improve energy efficiency. The idea presented in [14] was further explored for extension to mobility-based edge computing networks [15,16]. A total of *U* sensors share the *F* subchannels indexed by f∈F={1,2,…,F}.

However, there are three critical challenges to be addressed. Firstly, electromagnetic sensing data are transmitted over wireless channels and are therefore vulnerable to interception by potential eavesdroppers, resulting in serious data-security concerns. Moreover, most existing studies focus on task offloading for a single sensor or a single sensing device and do not adequately account for spectrum competition, inter-cell interference, and resource coupling arising from concurrent multi-sensor offloading. In addition, many existing works consider only the energy consumed by data transmission and computation, while neglecting the energy required for electromagnetic sensing and data acquisition. Consequently, they cannot accurately characterize the overall energy efficiency of the complete electromagnetic sensing, communication, and computing process.

### 1.2. Contributions

Motivated by the above issues, this work designs a data offloading framework for heterogeneous electromagnetic sensors, and then proposes a joint spectrum assignment, power and computing resource management scheme. The main contributions are summarized as follows.

- A secure edge-assisted data-offloading framework is developed for heterogeneous electromagnetic sensors deployed on IoT devices. Unlike existing studies that mainly consider a single sensor or a single type of sensing data, the proposed framework supports the concurrent acquisition and offloading of multiple types of electromagnetic sensing data. It further integrates sensing-data acquisition, secure wireless transmission, and edge processing into a unified IoT architecture.
- A secure energy efficiency (SEE) maximization problem is formulated for edge-assisted sensing-data transmission networks. The energy consumed by electromagnetic sensing, wireless transmission, and edge computing is jointly modeled with the end-to-end latency of sensing-data acquisition, offloading, and processing. Spectrum assignment, sensor power, and computing-resource allocation are jointly optimized to balance sensing-data security, energy consumption, and real-time processing requirements.
- An efficient joint resource-allocation strategy is proposed. Since the formulated mixed-integer optimization problem is non-convex and nondeterministic polynomial-time hard, a joint spectrum assignment, sensor power control, and computing-resource allocation algorithm is developed. The original problem is decomposed into a spectrum-assignment subproblem and a joint power-control and computing-resource-allocation subproblem. A modified Kuhn–Munkres method is employed for spectrum assignment, whereas convex optimization is used to obtain the transmit-power and computing-resource solutions. The subproblems are solved iteratively until convergence.
- The effectiveness of the proposed strategy is evaluated through simulations. The results demonstrate that the proposed method improves the SEE of secure electromagnetic sensing-data offloading under different system conditions.

## 2. System Model

### 2.1. Sensor Network Architecture

**Figure 1:**
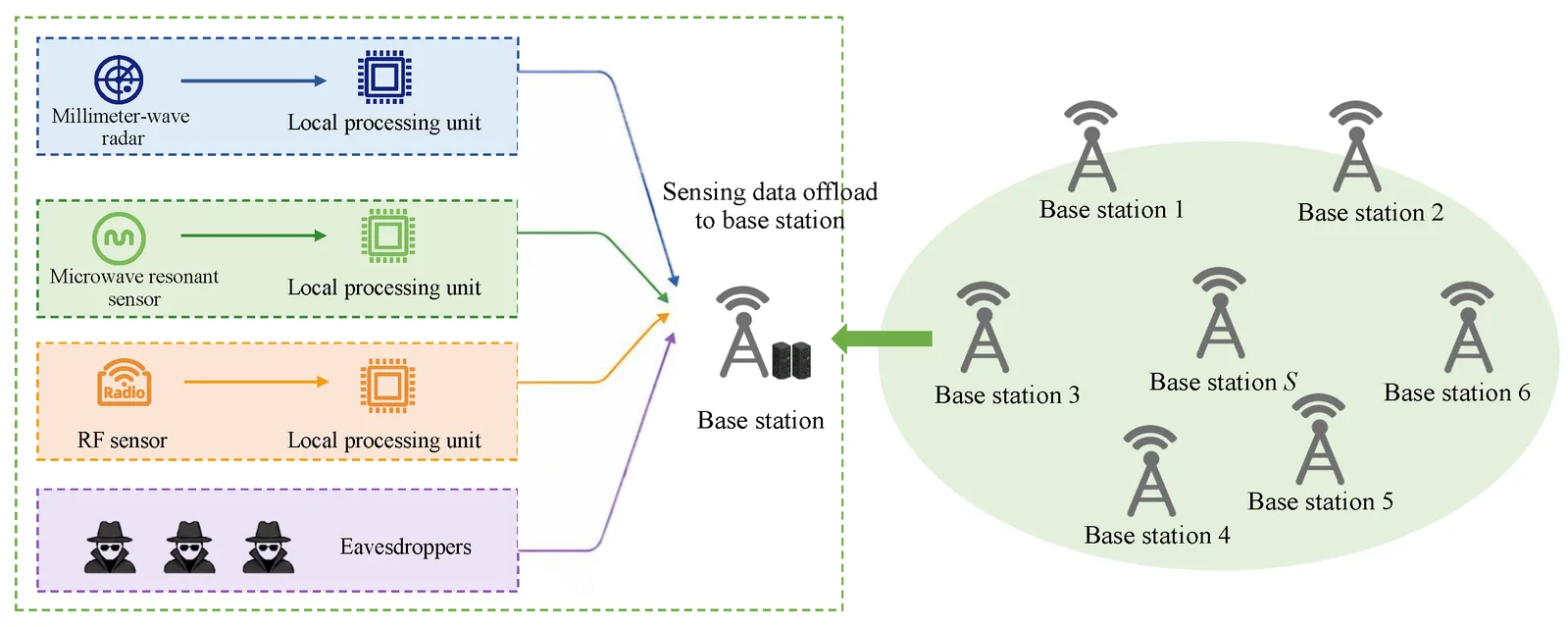
Sensor network architecture.

As illustrated in Figure 1, we consider an edge-assisted electromagnetic sensing network where each base station is equipped with an edge server. In the network, it consists of S base stations indexed by s∈S={1,2,…,S}, *U* sensors indexed by u∈U={1,2,…,U} and *E* eavesdroppers indexed by e∈E={1,2,…,E}. Each IoT device is equipped with an electromagnetic sensor, including millimeter-wave radar, microwave resonant sensor, radio-frequency imaging sensor, and impedance-based sensor. These sensors acquire electromagnetic responses associated with the physical environment, including range Doppler signatures, reflection coefficients, permittivity, conductivity, or impedance variations.

**Figure 2:**
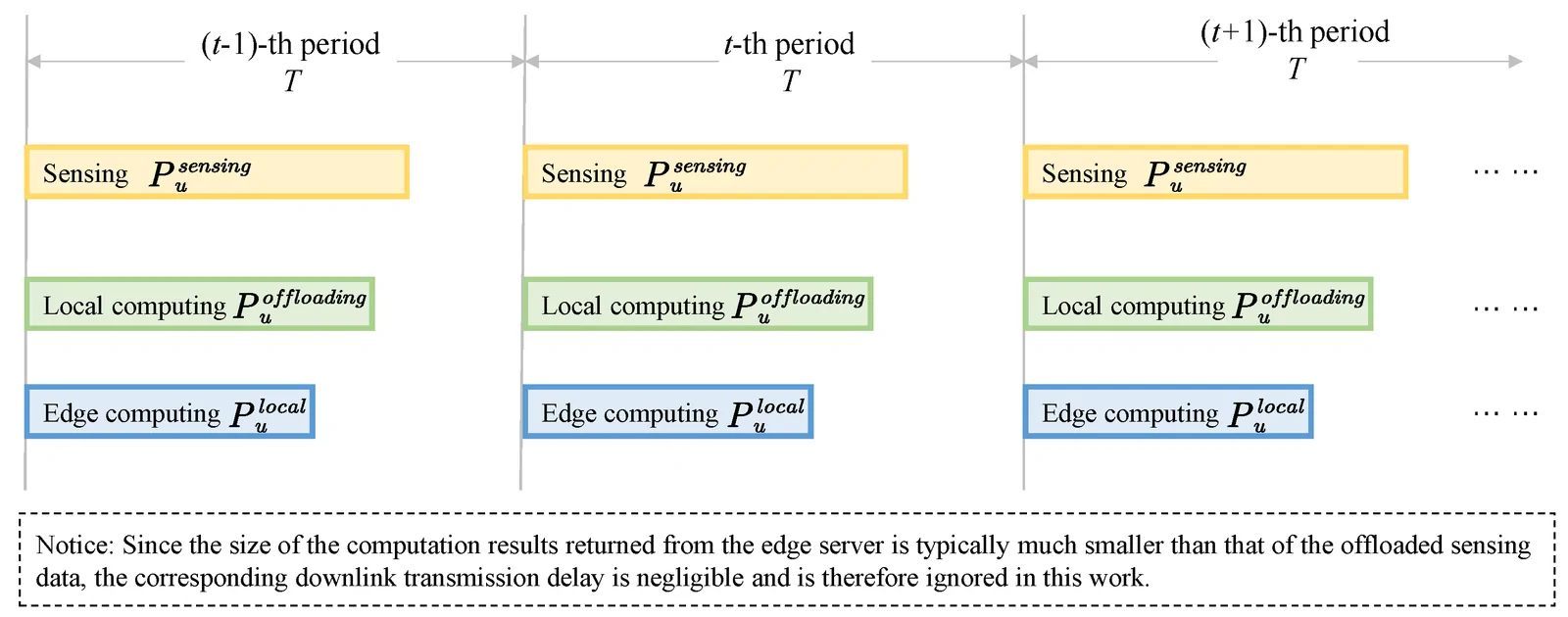
A diagram of the relationship between electromagnetic sensing and data offloading, where the data offloading process executes the data collected in the previous time period *T*.

Due to their limited battery capacity and computing capability, IoT devices cannot efficiently process computationally intensive electromagnetic sensing data locally. Therefore, the acquired sensing data are transmitted to the edge-based base station and processed by the edge server. This architecture establishes a combing electromagnetic sensing edge-computing, which can be shown in Figure 2. The system operates in a periodic manner with a period of *T*. Within each period *T*, two main operations are performed: (1) the sensing operation for the current period and (2) the offloading and computation of the sensing data collected in the previous period. The major notations are summarized in Table 1.

### 2.2. Electromagnetic Sensing-Data Model

Different electromagnetic sensors adopt distinct sensing mechanisms, resulting in heterogeneous sensing data with different acquisition rates, signal dimensions, computational complexities, and energy consumption characteristics. To establish a unified edge-computing framework, the sensing output generated by different electromagnetic sensors is abstracted into a common sensing-task model while preserving their heterogeneous characteristics. Each device is equipped with one electromagnetic sensor and periodically performs electromagnetic sensing during every sensing interval. Unlike conventional computation tasks, the sensing-data size depends on the sensing modality, sampling frequency, measurement duration, and quantization precision. Accordingly, the sensing-data size of sensor *u* can be expressed as

(1) $$D_{u}=f_{u}^{samp}T_{u}^{sensing}L_{u},$$

where $f_{u}^{samp}$ is the sampling frequency, $T_{u}^{sensing}$ is the sensing duration, and $L_{u}$ denotes the number of quantization bits for each sample.

### 2.3. Sensing-Data Offloading Model

During the processing of the sensing-data offloading, a total of *U* sensors share the *F* subchannels indexed by f∈F={1,2,…,F}. The signal-to-interference-plus-noise ratio (SINR) received by the *s*th base station from the *u*th sensor can be expressed as

(2) $$\gamma_{u,s}^{f}=\frac{P_{u}^{offloading}X_{u,f}{\left|G_{u,s}\right|}^{2}}{\sum_{u^{\prime }\neq u}P_{u^{\prime }}^{offloading}X_{u^{\prime },f}{\left|G_{u^{\prime },u}\right|}^{2}+\sigma^{2}},$$

where $P_{u}^{offloading}$ denotes the offloading power of the *u*th sensor. $\sigma^{2}$ is the noise power, $G_{u,s}$ indicates the channel gain of the *u*th device offloading their task to the *s*th BS. $X_{u,f}\in \left\{0,1\right\}$ denotes the spectrum assignment indicator. If the *u*th sensor associates with the *f*th SC, $X_{u,f}=1$; otherwise, $X_{u,f}=0$. Thus, the SINR received by the eavesdropper *e* from the *u*th sensor over the *f*th subchannel can be expressed as

(3) $$\gamma_{u,e}^{f}=\frac{P_{u}^{offloading}X_{u,f}{\left|{\hat{H}}_{u,e}\right|}^{2}}{\sum_{u^{\prime }\neq u}P_{u^{\prime }}^{offloading}{\left|{\hat{H}}_{u^{\prime },e}\right|}^{2}+\sigma^{2}}.$$

where ${\hat{H}}_{u,e}$ is the estimate channel gain obtained from long-term channel statistics between sensor *u* and eavesdropper *e* over the *f*th subchannel. The transmission rate and the best eavesdropping rate of the *u*th sensor can be respectively expressed as

(4) $$R_{u,s}^{f}=B_{0}\log_{2}\left(1+\gamma_{u,s}^{f}\right),$$

(5) $$R_{u,E}^{f}=\max_{e\in E}B_{0}\log_{2}\left(1+\gamma_{u,e}^{f}\right).$$

The secrecy rate, which is defined as the difference between transmission rates from the devices to the base station and that from the sensors to the eavesdropping, can be expressed as

(6) $$R_{u,s}^{sec}=\max\left(R_{u,s}^{f}-R_{u,E}^{f},0\right).$$

We define $C_{u}$ as the number of CPU cycles per bit, and $\beta_{u}$ as the proportion of the data allocated to edge server. The data processing delay at the *u*th sensor is

(7) $$\frac{D_{u}\left(1-\beta_{u}\right)C_{u}}{y_{u}}\leq T,$$

where $y_{u}$ (in CPU cycle/s) is the computing resource by the *u*th sensor. *T* is the maximum tolerable delay. The corresponding execution delay at the associated base station is

(8) $$\frac{D_{u}\beta_{u}}{R_{u,s}^{sec}}+\frac{D_{u}\beta_{u}C_{u}}{y_{u,s}}\leq T,$$

In this expression, $y_{u,s}$ denotes the computing resource allocated to user *u* by BS *s*, and *T* is the maximum tolerable delay. With a total computation capacity of $Y_{s}$ at BS *s*, the allocated resources must satisfy

(9) $$\sum_{u=1}^{U}y_{u,s}\leq Y_{s}.$$

The SEE of the studied networks is expressed as

(10) $$\xi =\frac{R_{total}}{P_{total}}=\frac{\sum_{u\in U}\sum_{s\in S}R_{u,s}^{sec}}{\sum_{u\in U}P_{u}^{offloading}+\sum_{u\in U}\sum_{s\in S}\kappa y_{u,s}^{3}},$$

where κ represents the effective capacitance coefficient of the CPU.

## 3. Secure Sensing-Data Offloading Problem Formulation

In this section, we formulate a SEE maximization problem for electromagnetic sensing-data offloading network by optimizing the sensing-data offloading power $P_{u}^{offloading}$, local CPU frequency $y_{u,s}$, and spectrum-allocation $X_{u,f}$, which is

(11a) $$\begin{array}{cc} \; & P1:\max_{y_{u,s},X_{u,f},P_{u}^{offloading}}\xi \end{array}$$

(11b) $$s.\;t.\;\;X_{u,f}\in \left\{0,1\right\},\forall u,f,$$

(11c) $$\begin{array}{cc} \; & 0\leq P_{u}^{sensing}+P_{u}^{offloading}+P_{u}^{local}\leq P_{u}^{max},\forall u, \end{array}$$

(11d) $$\begin{array}{cc} \; & \sum_{f\in F}X_{u,f}=1,\forall u, \end{array}$$

(7) − (9).(11e)

where $P_{u}^{max}$ is the maximum transmit power thresholds for the *u*th electronic sensor. Constraint (11c) lists feasible power. $P_{u}^{local}=\kappa y_{u}^{3}$. Constraint (11d) restricts that electronic sensor is assigned to only a single subchannel. (11a) is a fractional program and can be equivalently expressed in a subtractive form [17], yielding

(12) $$\max_{y_{u,s},X_{u,f},P_{u}^{offloading}}\left(R_{total}-\xi^{*}P_{total}\right).$$

The optimization problem is NP-hard and is therefore decomposed into two separate subproblems: (1) the binary variable optimization subproblem (i.e., spectrum resource optimization subproblem) and (2) the continuous variable optimization subproblem (electromagnetic-sensor power and computation resource optimization subproblem), which is tackled in the next section.

## 4. Solution of the SEE Optimization Problem

### 4.1. Spectrum Allocation Decision

By fixing the electromagnetic-sensor power and computation resource decisions, the original optimization issue is reduced to

(13a) $$\begin{array}{cc} \; & P2:\max_{X_{u,f}}\left(R_{total}-\xi^{*}P_{total}\right) \end{array}$$

(13b) $$s.\;t.\;\;X_{u,f}\in \left\{0,1\right\},\forall s,f,$$

(13c) $$\begin{array}{cc} \; & \sum_{f\in F}X_{u,f}=1,\forall s, \end{array}$$

Obviously, P2 is a non-convex optimization problem. To solve it, we design a revised Kuhn–Munkres-based matching algorithm, which can be separated into two parts. In the first stage, a weighted bipartite graph, denoted by G=(A,B,W), is constructed. Here, A and B represent the vertex sets corresponding to the sensors and subchannels, respectively, while W denotes the associated weight matrix. The weight assigned to the edge connecting sensor vertex $A_{u}$ and subchannel vertex $B_{f}$ is expressed as

(14) $$W_{A_{u},B_{f}}=R_{u,s}^{sec}/\left(P_{u}^{offloading}+\kappa y_{u,s}^{3}\right),\;\forall A_{u}\in A,B_{f}\in B.$$

**Figure 3:**
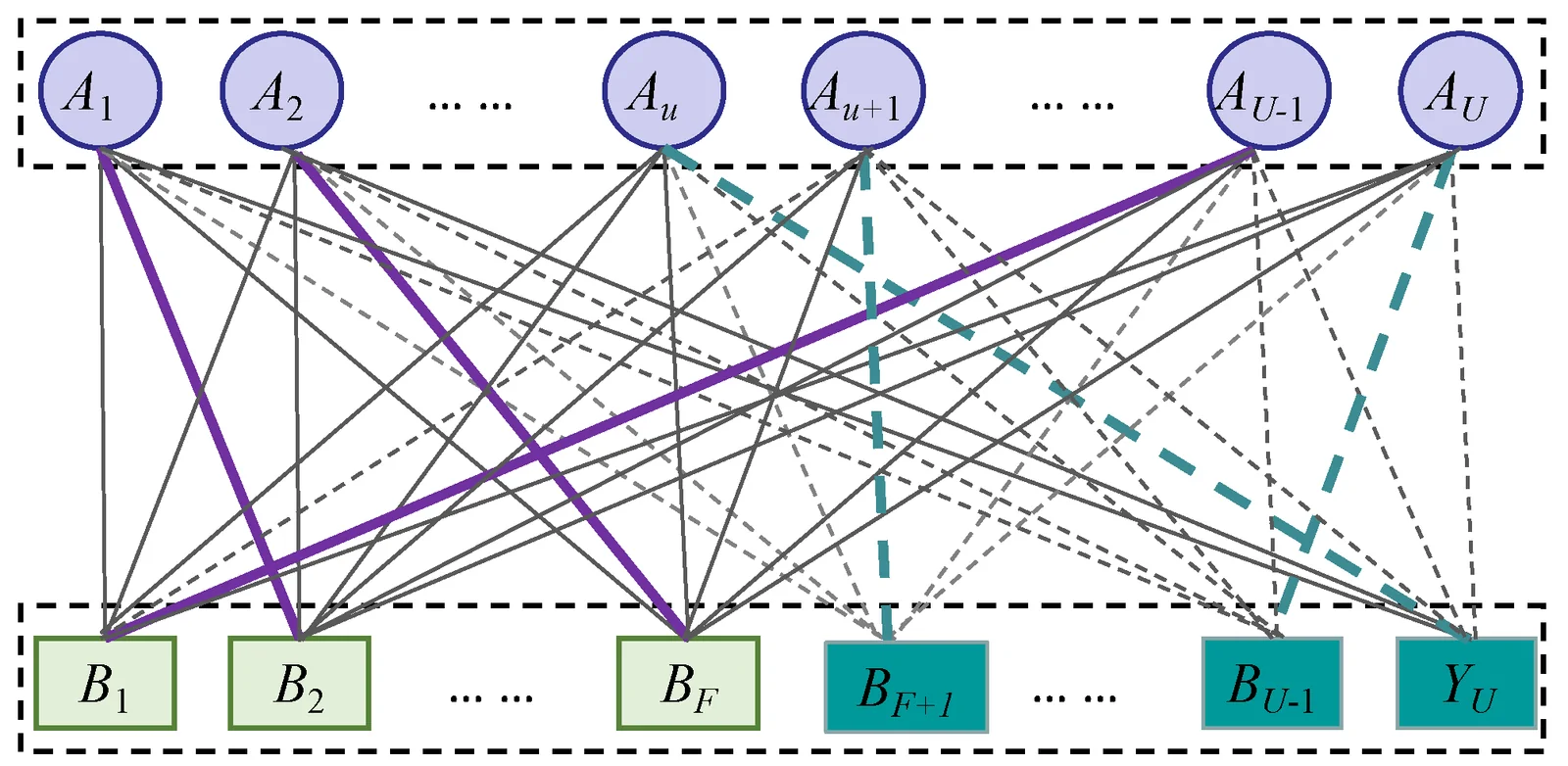
An illustration of the revised Kuhn–Munkres matching algorithm.

The second stage performs the matching using a modified Kuhn–Munkres algorithm. The conventional Kuhn–Munkres algorithm is applicable to a balanced bipartite graph in which the two vertex sets have the same cardinality [18]. In the considered system, however, the number of sensors may be larger than the number of available subchannels. To address this mismatch, U−F dummy vertices are introduced and connected through zero-weight edges. In this manner, the original graph G is transformed into the balanced virtual graph $G^{\prime }$, as illustrated in Figure 3. The resulting graph can then be processed using the Kuhn–Munkres method. The key index in Kuhn–Munkres algorithm is the feasible vertex labeling which is defined in Definition 1.

**Definition** **1.** *A feasible vertex label in $G^{\prime }$ can be defined as*

(15) $$\begin{array}{c} \left\{\begin{array}{c} L_{B_{u}}=\max\left\{W_{B_{u},Y_{m}}\right\},\forall Y_{m}\in Y,\;\mathrm{if}\;B_{u}\in B,\\ L_{Y_{m}}=0,\;\mathrm{if}\;Y_{m}\in Y. \end{array}\right. \end{array}$$

### 4.2. Electromagnetic-Sensor Power Control and Computing Decision

After obtained the spectrum assignment decision, the problem can be converted into the electromagnetic-sensor power and computation resource optimization subproblem, which can be further converted into P3.1, a computation resource allocation subproblem, and P3.2, an electromagnetic-sensor power control subproblem.

(16a) $$\begin{array}{cc} & P3.1:\max_{y_{u,s}}\left(R_{total}-\xi^{*}P_{total}\right) \end{array}$$

(16b) $$s.\;t.\;\;\frac{D_{u}\beta_{u}}{R_{u,s}^{sec}}+\frac{D_{u}\beta_{u}C_{u}}{y_{u,s}}\leq T,\forall u,s,$$

(16c) $$\begin{array}{cc} \; & \sum_{u=1}^{U}y_{u,s}\leq Y_{s},\forall s. \end{array}$$

(17a) $$\begin{array}{cc} & P3.2:\max_{P_{u}^{offloading}}\left(R_{total}-\xi^{*}P_{total}\right) \end{array}$$

(17b) $$s.\;t.\;\;0\leq P_{u}^{offloading}\leq P_{u}^{th},\forall u,$$

where $P_{u}^{th}=P_{u}^{max}-P_{u}^{sensing}-P_{u}^{local}$. According to the definition of convex function in [19], P3.1 is convex and solvable using standard convex optimization approaches. Using the logarithmic function property, (17a) can be rewritten as

(18) $$\begin{array}{cc} \; & \max_{{P_{u}}^{offloading}}\sum_{u\in U}B_{0}(\underset{\delta_{1}}{\underset{︸}{\log_{2}\left(P_{u}^{offloading}{\left|G_{u,s}\right|}^{2}+\sum_{u^{\prime }\neq u}P_{u^{\prime }}^{offloading}{\left|G_{u^{\prime },u}\right|}^{2}+\sigma^{2}\right)}}-\Phi \left(\tilde{P}\right)\\ \; & +\underset{\delta_{2}}{\underset{︸}{\log_{2}\left(\sum_{u^{\prime }\neq u}P_{u^{\prime }}^{offloading}{\left|{\hat{H}}_{u^{\prime },E}\right|}^{2}+\sigma^{2}\right)}}-\Psi \left(\tilde{P}\right))-\xi^{*}\sum_{u\in U}\left(P_{u}^{offloading}+\kappa y_{u,s}^{3}\right) \end{array}$$

where $\Phi \left(\tilde{P}\right)=\log_{2}\left(\sum_{u^{\prime }\neq u}P_{u^{\prime }}^{offloading}{\left|G_{u^{\prime },u}\right|}^{2}+\sigma^{2}\right)$, $\Psi \left(\tilde{P}\right)=\log_{2}\left(P_{u}^{offloading}{\left|{\hat{H}}_{u,E}\right|}^{2}+\sum_{u^{\prime }\neq u}P_{u^{\prime }}^{offloading}{\left|{\hat{H}}_{u^{\prime },E}\right|}^{2}+\sigma^{2}\right)$. Since $\Phi (\tilde{P})$ and $\Psi (\tilde{P})$ are logarithms of positive affine functions of $\tilde{P}$, both functions are concave. Therefore, their first-order Taylor expansions at ${\tilde{P}}^{\left(k\right)}$ provide global upper bounds, i.e.,

(19) $$\Phi \left(\tilde{P}\right)\leq \Phi \left({\tilde{P}}^{\left(k\right)}\right)+\nabla \Phi {\left({\tilde{P}}^{\left(k\right)}\right)}^{T}\left(\tilde{P}-{\tilde{P}}^{\left(k\right)}\right),$$

and

(20) $$\Psi \left(\tilde{P}\right)\leq \Psi \left({\tilde{P}}^{\left(k\right)}\right)+\nabla \Psi {\left({\tilde{P}}^{\left(k\right)}\right)}^{T}\left(\tilde{P}-{\tilde{P}}^{\left(k\right)}\right).$$

Accordingly, the corresponding negative terms are replaced by affine lower bounds, yielding a concave surrogate objective that is tight at the current point ${\tilde{P}}^{\left(k\right)}$. Hence, P3.2 can be approximated by P3.2′ as

$$\begin{array}{cc} {P3.2}^{\prime }:\; & \max_{P_{u}^{offloading}}\sum_{u\in U}B_{0}\left(\delta_{1}-\hat{\Phi }\left(\tilde{P},{\tilde{P}}^{\left(k\right)}\right)+\delta_{2}-\hat{\Psi }\left(\tilde{P},{\tilde{P}}^{\left(k\right)}\right)\right) \end{array}$$

(21a) $$\begin{array}{cc} \; & -\xi^{*}\sum_{u\in U}\left(P_{u}^{\mathrm{offloading}}+\kappa y_{u,s}^{3}\right) \end{array}$$

(21b) $$s.\;t.\;\;0\leq P_{u}^{offloading}\leq P_{u}^{th},\forall u,$$

where $\hat{\Phi }\left(\tilde{P},{\tilde{P}}^{\left(k\right)}\right)=\Phi \left({\tilde{P}}^{\left(k\right)}\right)+\nabla \Phi {\left({\tilde{P}}^{\left(k\right)}\right)}^{T}\left(\tilde{P}-{\tilde{P}}^{\left(k\right)}\right),$ $\hat{\Psi }\left(\tilde{P},{\tilde{P}}^{\left(k\right)}\right)=\Psi \left({\tilde{P}}^{\left(k\right)}\right)+\nabla \Psi {\left({\tilde{P}}^{\left(k\right)}\right)}^{T}\left(\tilde{P}-{\tilde{P}}^{\left(k\right)}\right)$. Since $\delta_{1}$ and $\delta_{2}$ are concave functions of the transmit-power variables, while $\hat{\Phi }$ and $\hat{\Psi }$ are affine, the objective function of P3.2′ is concave. Moreover, the feasible set defined by the power constraints is convex. Therefore, P3.2′ is a convex optimization problem and can be efficiently solved using CVX [20]. The approximation is updated iteratively until convergence.

### 4.3. Joint Optimization Algorithm Description

The SEE maximization problem P1 is solved via a two-stage decomposition framework. Spectrum assignment is first optimized using Kuhn–Munkres algorithm, followed by joint electromagnetic-sensor power control and computation resource allocation under the fixed spectrum. The whole algorithm is summarized in Algorithm 1. For the spectrum assignment subproblem P2, we adopt Kuhn–Munkres algorithm. The complexity can be approximated to be $O(\chi^{3})$, where χ is max{U,F}. Since $F\ll \chi^{3}$, the complexity at this stage approximates to $O(L_{2}\chi^{3})$,where the iteration number is $L_{2}$. For the joint power and computing decision subproblem, CVX is used to solve the subproblems P3.1 and P3.2. Since CVX invokes the interior-point method to solve optimization problems, the complexity of solving joint power and computing decision subproblem is $O({\left(US+S\right)}^{\frac{1}{2}}\left(2US+S\right)U^{2}S^{2}+ℜ)$, where $ℜ={\left(2U+US\right)}^{\frac{1}{2}}\left(3U+US\right)U^{2}$. Summarizing all above, the complexity of the whole algorithm can be expressed as $O(L_{1}\left(L_{2}\chi^{3}+{\left(US+S\right)}^{\frac{1}{2}}\left(2US+S\right)U^{2}S^{2}+ℜ\right))$ with $L_{1}$ denoting the required number of outer iterations.
**Algorithm 1** Joint Optimization Algorithm1:**Input:**Maximum iteration number Iter=1000 and the maximum tolerance $\epsilon =10^{-4}$.2:**Output:**SEE value $\xi^{*}$, spectrum assignment decision $X_{s,f}^{*}$, computing resource decision $y_{u,s}^{*}$ and transmit power decision $P_{u}^{*}$.3:Initialize $\xi^{\left(0\right)}=0$, $flag_{\xi }=1$, flag=1, outer iteration index t=0 and δ=0.4:**while** $flag_{\xi }>\epsilon$ and t<Iter **do**5:   t=t+1.6:   **while** flag>ϵ and δ<Iter **do**7:      δ=δ+1.8:      Given power control and computing decision, perform the modified Kuhn–Munkres method to obtain the spectrum assignment decision $X_{s,f}^{\left(\delta \right)}$.9:      Under a fixed spectrum assignment, joint power and computing resource allocation is executed. Calculate $y_{u,s}^{\left(\delta \right)}$ and $P_{u}^{\left(\delta \right)}$.10:     Calculate $flag=\max_{u}\left|P_{u}^{\left(\delta \right)}-P_{u}^{\left(\delta -1\right)}\right|$.11:  **end while**12:  Calculate $flag_{\xi }=\left|R_{total}\left(X_{s,f}^{\left(\delta \right)},y_{u,s}^{\left(\delta \right)},P_{u}^{\left(\delta \right)}\right)-\xi^{\left(s-1\right)}P_{total}\left(P_{u}^{\left(\delta \right)}\right)\right|$.13:  Update $\xi^{\left(s\right)}$ via $\xi^{\left(s\right)}=\frac{=R_{total}\left(X_{s,f}^{\left(\delta \right)},y_{u,s}^{\left(\delta \right)},P_{u}^{\left(\delta \right)}\right)}{P_{total}\left(P_{u}^{\left(\delta \right)}\right)}$.14:**end while**15:Calculate $X_{s,f}^{*}=X_{s,f}^{\left(\delta \right)}$, $y_{u,s}^{*}=y_{u,s}^{\left(\delta \right)}$ and $P_{u}^{*}=P_{u}^{\left(\delta \right)}$.16:Calculate $\xi^{*}=\frac{R_{total}\left(X_{s,f}^{*},y_{u,s}^{*},P_{u}^{*}\right)}{P_{total}\left(P_{u}^{*}\right)}$.

## 5. Simulation Results and Analysis

IoT devices equipped with heterogeneous electromagnetic sensors and passive eavesdroppers are randomly distributed within a 1000 m × 1000 m area. Nine base stations, each integrated with an edge computing server and having a coverage radius of 100 m, are deployed in this area to support electromagnetic sensing-data offloading. The total number of IoT sensing devices varies from 20 to 240 to evaluate the scalability of the proposed scheme under different sensing-device densities. These devices are modeled as heterogeneous sensing nodes, such as millimeter-wave radar and microwave sensing devices, and therefore generate sensing tasks with different data sizes, computational workloads, latency requirements, and sensing-energy consumption. Passive eavesdroppers are randomly located within the considered area and attempt to intercept the sensing data transmitted from the IoT devices to the base stations.In addition, each data point presented in the simulation figures is obtained by averaging the results over 1000 independent Monte Carlo realizations. The channel parameters are configured following [17]. The large-scale path loss for both the sensor-to-BS and sensor-to-eavesdropper links is modeled as 128.1+37.6lg(d) dB, where *d* is the corresponding link distance in kilometers. Small-scale fading is modeled as independent Rayleigh fading, where the channel coefficient follows h∼CN(0,1). The remaining simulation parameters are summarized in Table 2.

**Figure 4:**
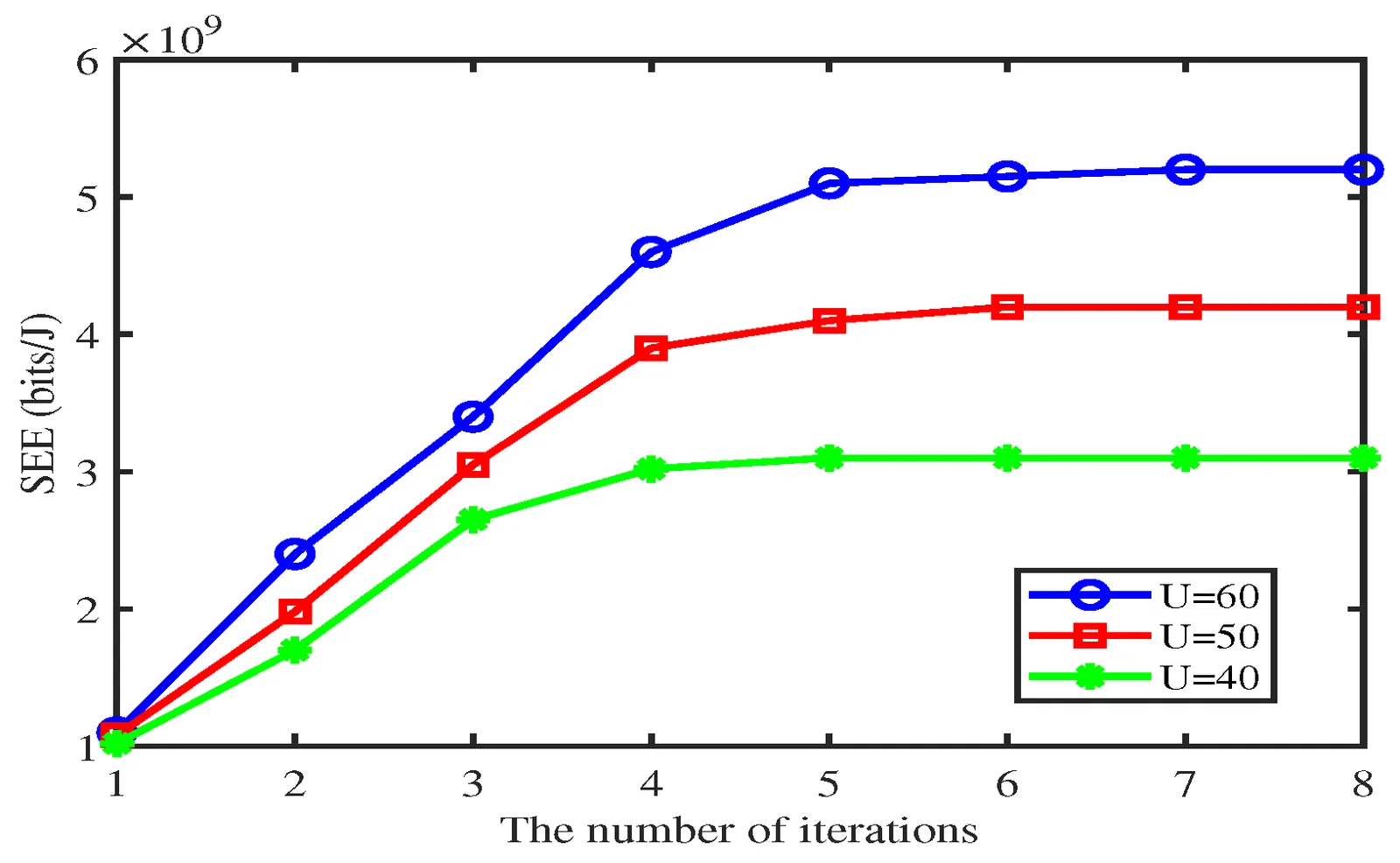
Convergence analysis of the proposed algorithm.

To verify the feasibility of the proposed algorithm, this work firstly evaluate its convergence performance. The convergence performance of the proposed algorithm for varying device numbers is presented in Figure 4, where $P_{u}^{max}=23$ dBm, $D_{u}=10^{4}$ bits, T=400 ms and F=50. The proposed scheme converges within seven iterations for different numbers of sensors. When the number of sensors is larger, the convergence time can be increased. This is because that the increasing number of sensors leads to an increasing searching space, which in turn increases the number of iterations. In addition, the energy efficiency of the sensor network increases as the number of subchannels. The reason is that the enhancement in bandwidth resources mitigates co-channel interference between sensors, leading to an improved secure offload rate with the given transmit power.

Furthermore, we studied the impact of the parameters (the number of sensors, the number of active base stations, the distance from eavesdropper to central area, the computational capacity of sensor, the size of offloaded data and the maximum tolerant delay of each sensor) on the energy efficiency performance. The proposed algorithm is compared with three baseline algorithms through simulations using the same simulation parameters.

(1) **IntI-EE:** This algorithm proposed in [21] includes inter-cell interference management strategy and does not consider the security of task offloading.
(2) **InnI-SEE:** This algorithm proposed in [22] considers the security of task offloading and does not manage inter-cell interference.
(3) **InnI-EE:** This algorithm proposed in [23] does not manage inter-cell interference and not consider the security of task offloading.

**Figure 5:**
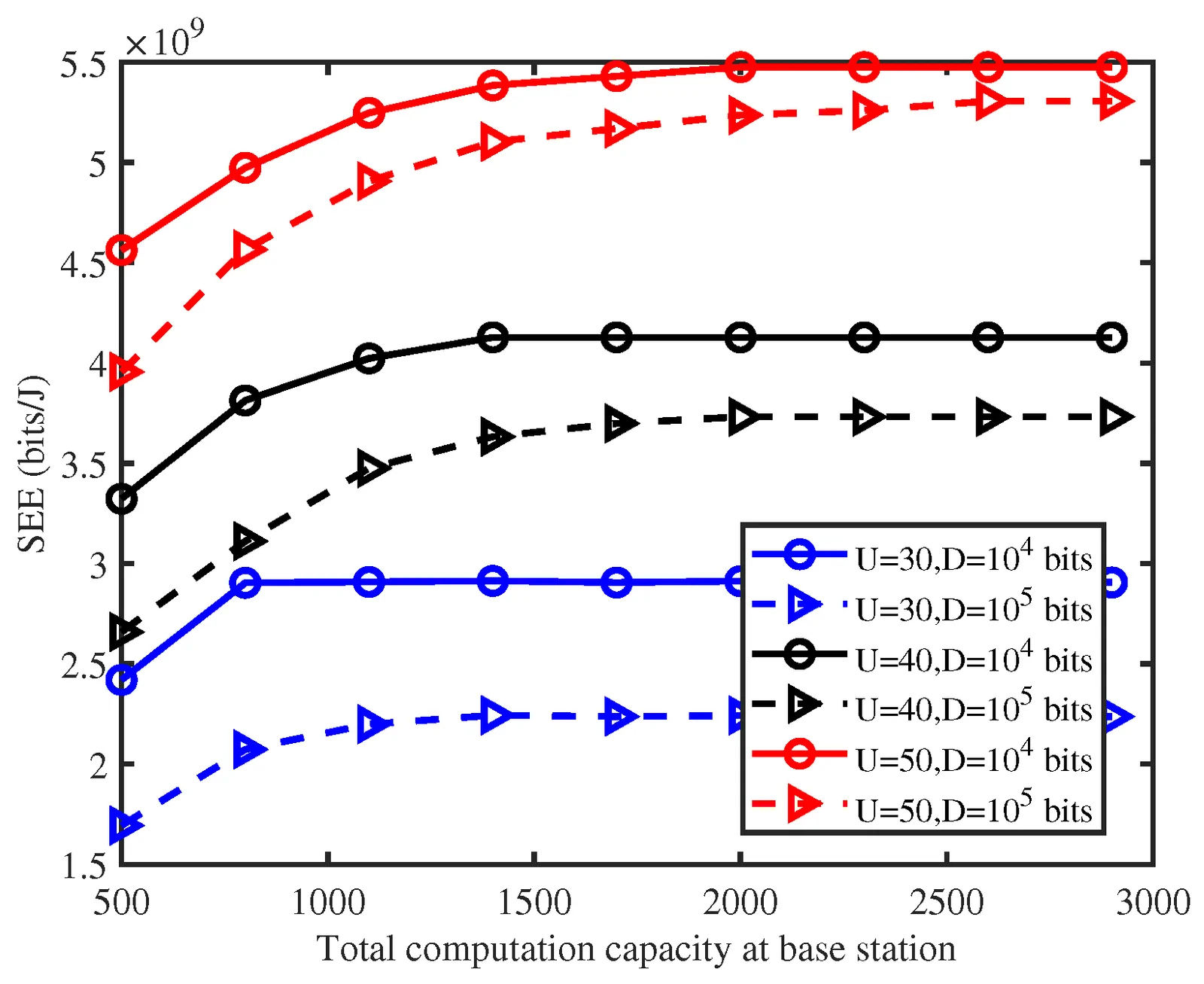
The secure energy efficiency for different computation capacities at each base station.

Figure 5 shows the effect of total computation capacity at each base station on SEE with $P_{u}^{\max}=20$ dBm, F=50, and T=400 ms. While with the number of sensors and sensing data keep fixed, the SEE of the proposed algorithm initially increases with the increment of total computation capacity before stabilizing, indicating the existence of a critical value of total computation capacity. This critical value represents the minimum number of total computation capacity required to meet the high SEE demands of devices. In addition, with the increase in the number of sensors and the sensing data, the critical value of total computation capacity also rises, which suggests that the total computation capacity assigned to each MEC server should be determined based on the number of served sensors and the volume of data.

**Figure 6:**
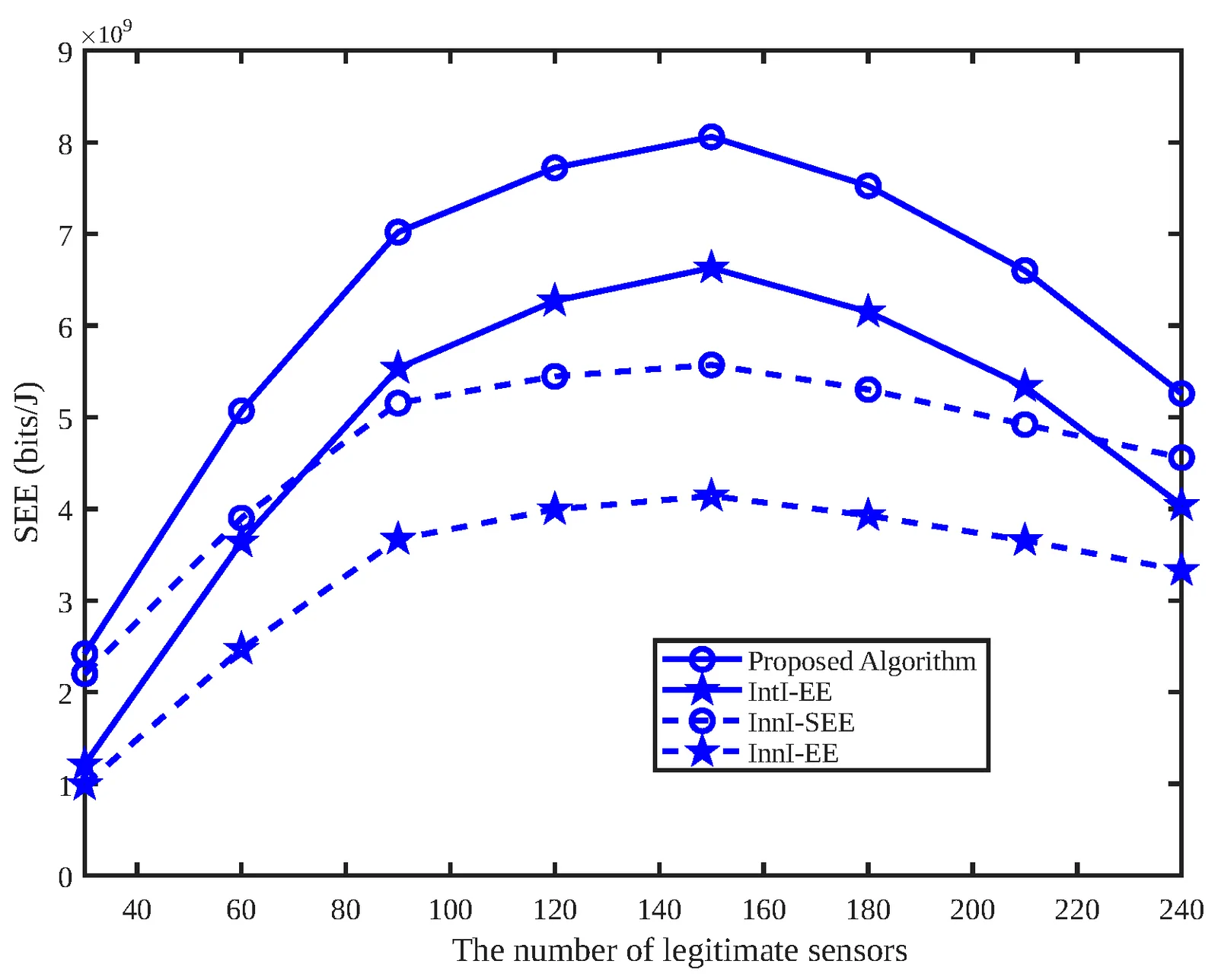
The secure energy efficiency for different number of legitimate sensors.

The impact of the number of sensors at each base station on the energy efficiency is simulated by setting $P_{u}^{max}=20$ dBm, F=50, $D_{u}=10^{4}$ bits, and T=400 ms, and the obtained results are shown in Figure 6. As shown in Figure 6, our proposed scheme achieves higher SEE than baseline algorithms. We also observe that at higher sensor densities, schemes that do not consider inter-cell interference management, namely InnI schemes, do not incur significant performance degradation. Moreover, when disregarding the issue of secure offloading, and with sensor numbers at 120, the performance loss reaches 1.6×10 bits/J. In other words, with 120 sensors, the proposed scheme outperforms the IntI-EE scheme by approximately 25% in terms of SEE performance.

**Figure 7:**
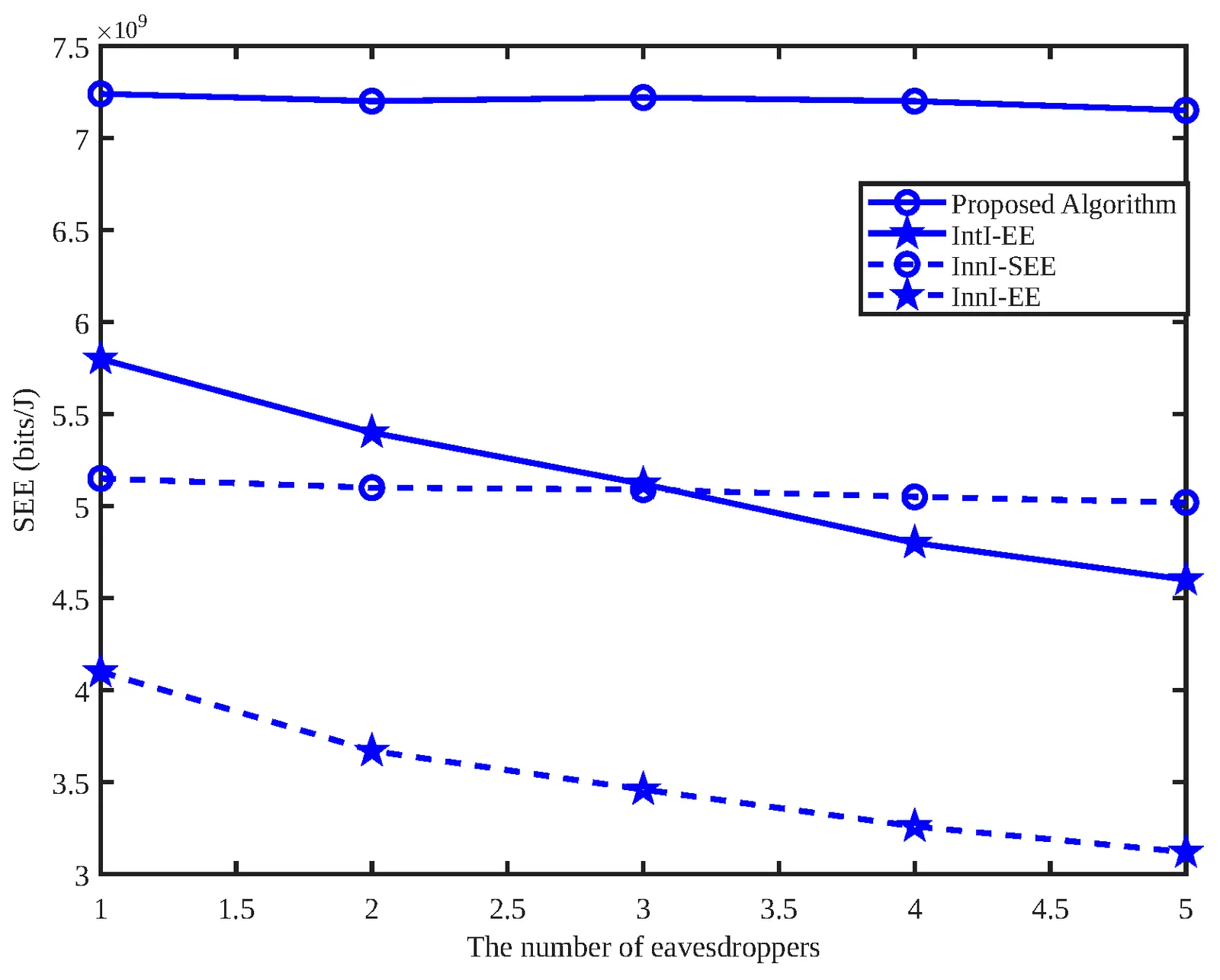
The secure energy efficiency for different number of eavesdroppers.

**Figure 8:**
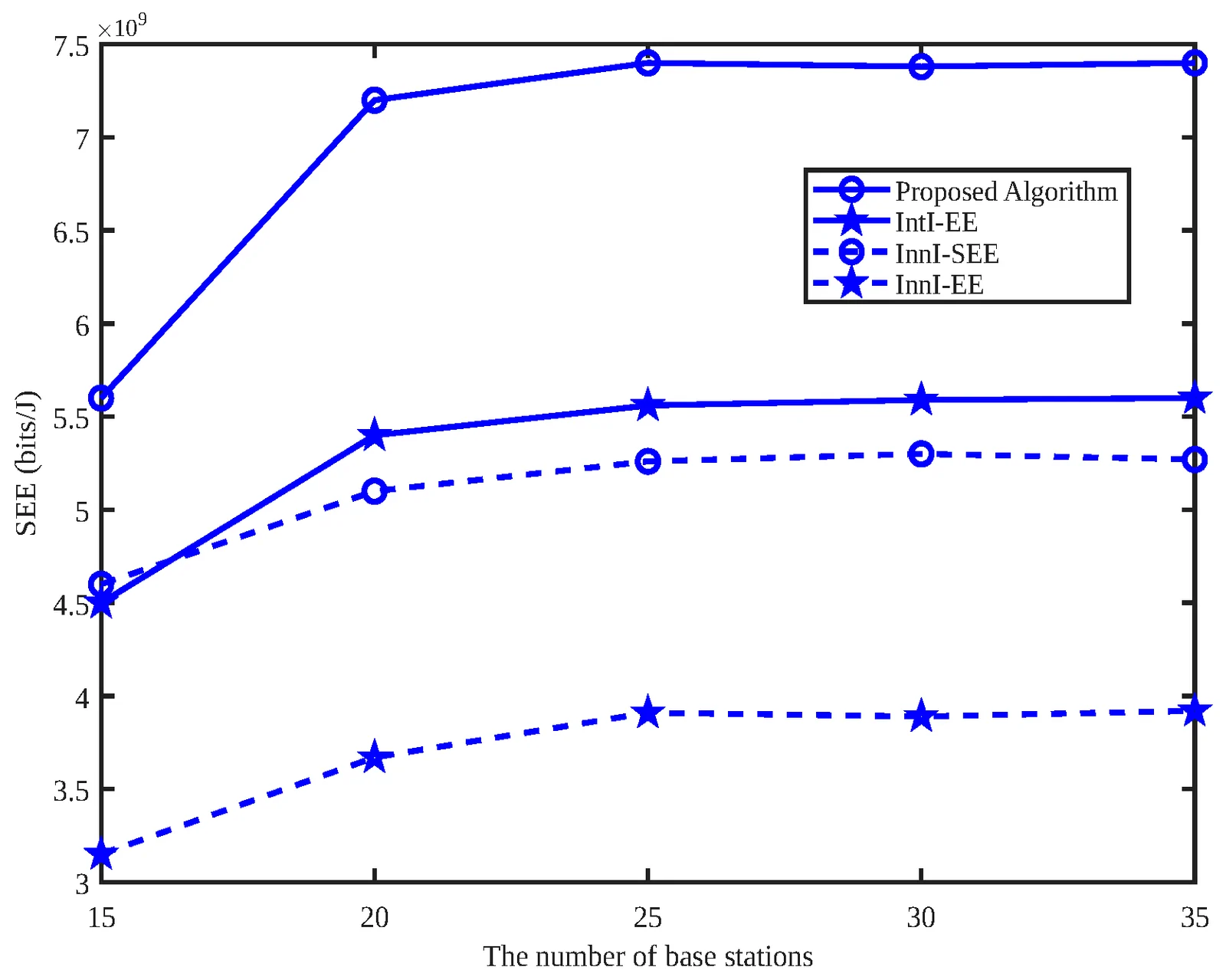
The secure energy efficiency for different network base station deployment densities.

To investigate the influence of eavesdropper quantity on the SEE, we set $P_{u}^{max}=20$ dBm, F=50, $D_{u}=10^{4}$ bits, and T=400 ms, and the obtained results are shown in Figure 7. With the increase in eavesdroppers, the SEE-driven algorithm exhibits strong resistance to eavesdropping, whereas the anti-eavesdropping performance of other benchmark algorithms decreases significantly. In particular, the proposed algorithm achieves the highest resistance to eavesdropping, further demonstrating its superiority. The effect of varying the base station deployment density on SEE is evaluated, as shown in Figure 8, where $P_{u}^{max}=20$ dBm, F=50, $D_{u}=10^{4}$ bits, and T=400 ms. As shown in the figure, the proposed algorithm outperforms the others in terms of SEE. While the number of deployed base stations is fewer than 25, the SEE performance of all algorithms improves with the increase in deployment density, primarily due to the gains brought by denser base station deployment. However, as the deployment density continues to increase, the marginal gains become negligible. At this stage, the limiting factor for SEE improvement is no longer the base station deployment density.

**Figure 9:**
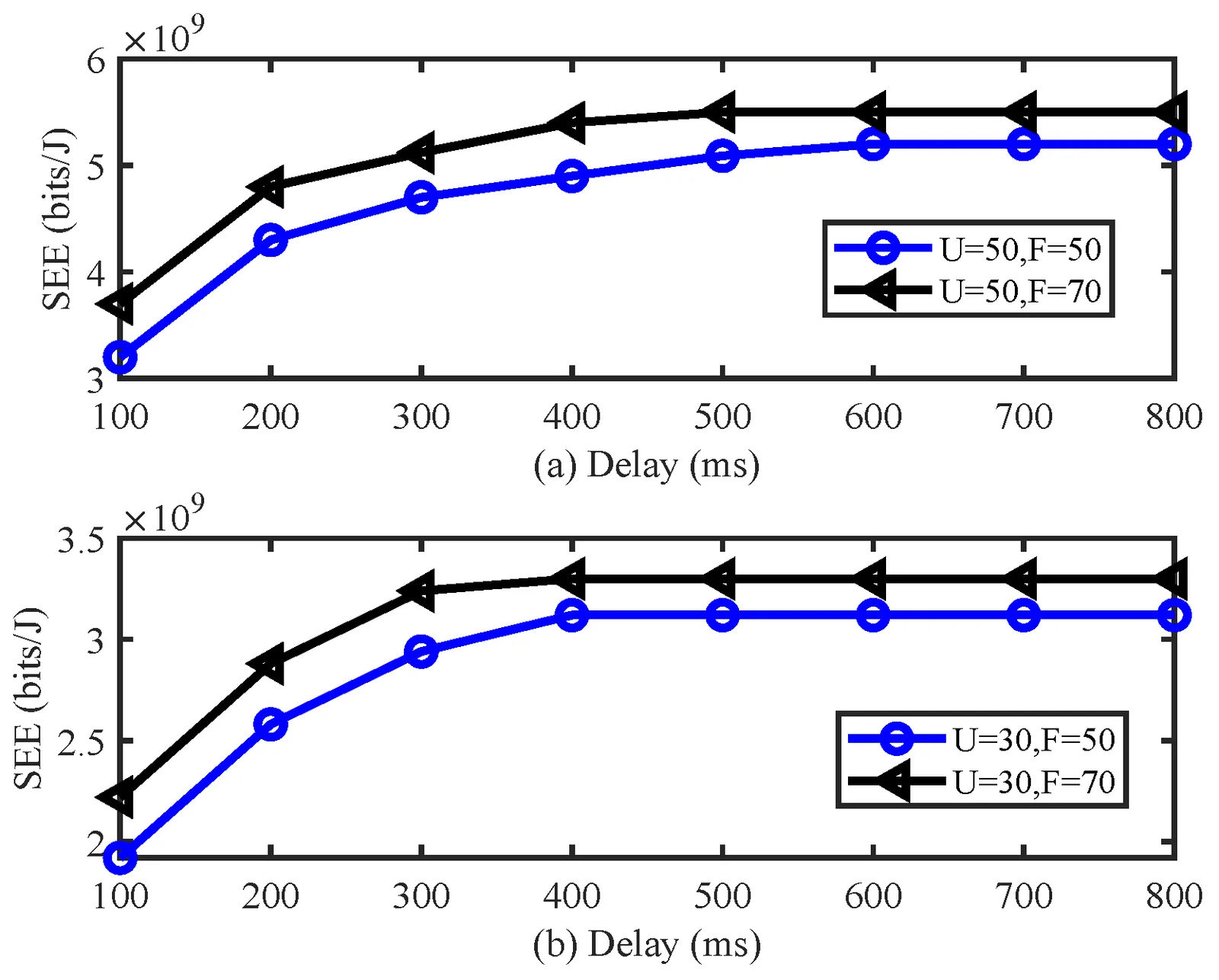
The secure energy efficiency with different delays. (**a**) U = 50, (**b**) U = 30.

The impact of the maximum tolerant delay of each sensor on SEE is simulated by setting $P_{u}^{max}=20$ dBm, F=50, $D_{u}=10^{4}$ bits. The results are included in Figure 9, which shows that enhancing the maximum tolerant delay of each device can improve the SEE. With the number of sensors and subchannels fixed, there exists a critical value for the maximum delay. When the tolerant delay exceeds the critical value, the maximum tolerant delay is no longer the primary factor limiting SEE. SEE performance is constrained by limited communication and computational resources. Furthermore, fixed subchannels and devices, with an increase in total computational resources, more resources are allocated to devices, enhancing the system SEE.

**Figure 10:**
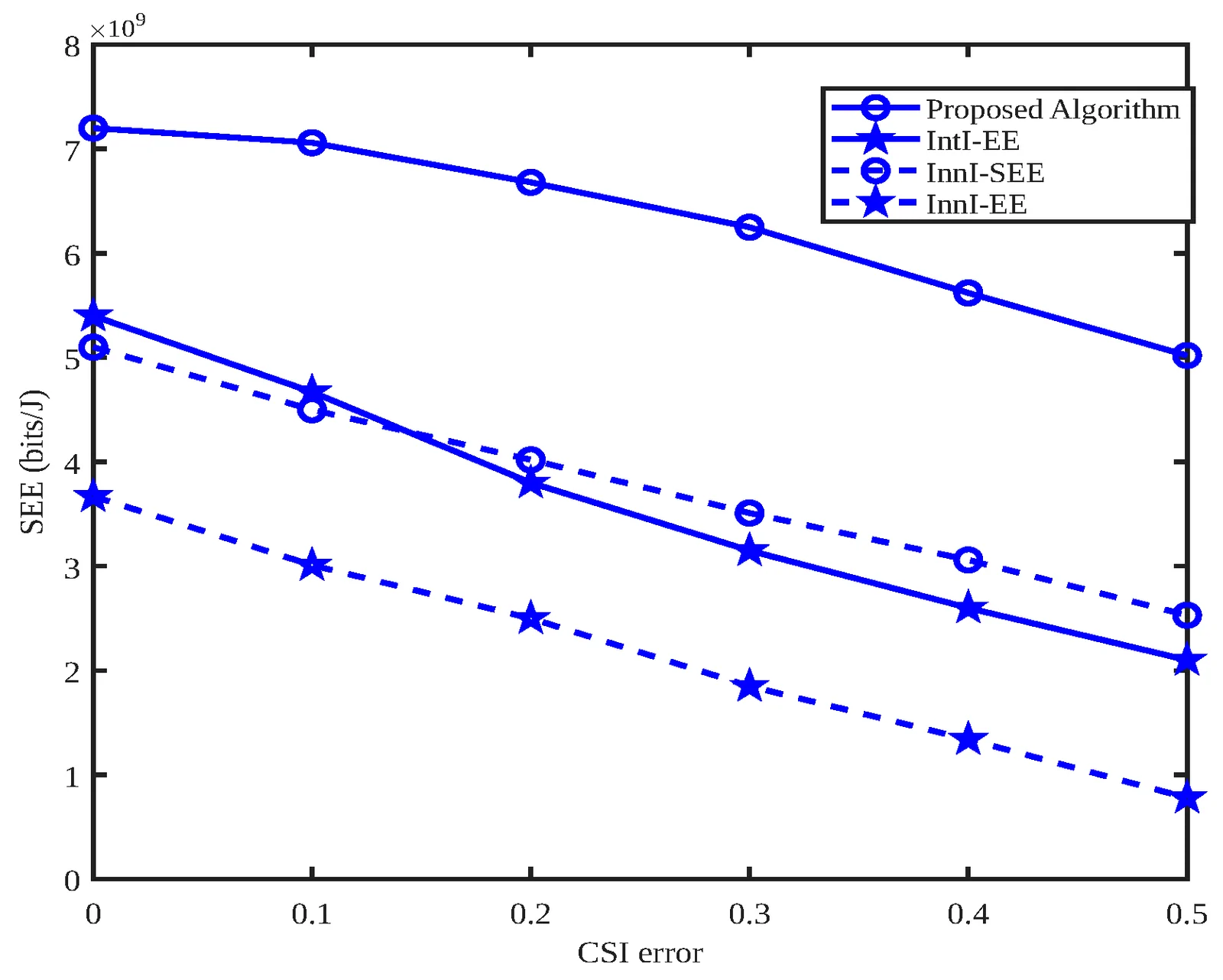
The secure energy efficiency with different CSI errors.

Figure 10 analyzes the SEE performance under both perfect and imperfect CSI, where δ denotes the CSI estimation error and δ=0 denotes the perfect CSI. As δ increases from 0 to 50%, the SEE achieved by the proposed algorithm decreases by 28%, as illustrated in Figure 10. Clearly, with larger channel estimation errors, the error allocation rate of communication and computation resources increases, making it more difficult to satisfy the requirements of secure task offloading, thereby degrading the SEE performance. Compared with other benchmark algorithms, the proposed algorithm achieves an SEE gain of at least 31%.

## 6. Conclusions

This work investigated secure and energy-efficient sensing-data offloading in IoT networks comprising heterogeneous electromagnetic sensors. To support real-time processing while protecting sensitive sensing data from eavesdropping, a secrecy energy efficiency maximization problem was formulated by jointly optimizing spectrum assignment, transmit power, and edge computing-resource allocation under resource constraints. The proposed formulation accounts for the energy consumed by electromagnetic sensing and edge computing, thereby providing a comprehensive framework for evaluating secure electromagnetic sensing-data offloading. The simulation results demonstrated that the proposed algorithm consistently improves secrecy energy efficiency and achieves an SEE gain of at least 31% over the benchmark schemes within a specific range. These results confirm the effectiveness of the proposed framework for secure, low-latency, and energy-efficient offloading of heterogeneous electromagnetic sensing data in edge-assisted IoT networks.

## Figures and Tables

**Table 1:** Notations.

Notations	Descriptions
*s*	base station
*u*	sensor
*e*	eavesdropper
*f*	subchannel
$\gamma_{u,.s}^{f}$	SINR from sensor to base station
$\gamma_{u,.E}^{f}$	SINR from sensor to eavesdropper
$\beta_{u}$	proportion of data allocated to edge server
$y_{u}$	computing resource by the sensor locally
$y_{u,s}$	computing resource allocated to sensor by base station
$X_{u,f}$	spectrum allocation
$P_{u}^{offloading}$	sensing-data offloading power

**Table 2:** Simulation parameters and values.

Parameters	Values
Maximum number of base stations	35
Number of eavesdroppers	[1, 5]
Offloading ratio $\beta_{u}$	0.6
Maximum iterations	1000
Convergence tolerance	$10^{-4}$
Path-loss model	128.1+37.6lg(d) dB, *d* in km
Small-scale fading model	Rayleigh fading
Maximum transmit power of each electromagnetic-sensor $P_{u}^{max}$	[20, 26] dBm
Subchannel bandwidth $B_{0}$	180 kHz
Carrier frequency	2 GHz
Noise power $\sigma^{2}$	−104 dBm
Number of subchannels *F*	[50, 70]
Size of offloaded data $D_{u}$	$\left[10^{4},10^{5}\right]$ bits
Number of CPU cycles for computing 1 bit data $C_{u}$	$10^{4}$ cycle/bit
Maximum tolerant delay *T* [17]	[100,800] ms
Capacitance coefficient κ [17]	$10^{-20}$

## Data Availability

The data provided in this study can be obtained from the corresponding author.
